# Inorganic Biomaterials Shape the Transcriptome Profile to Induce Endochondral Differentiation

**DOI:** 10.1002/advs.202402468

**Published:** 2024-05-13

**Authors:** Aparna Murali, Anna M. Brokesh, Lauren M. Cross, Anna L. Kersey, Manish K. Jaiswal, Irtisha Singh, Akhilesh Gaharwar

**Affiliations:** ^1^ Department of Biomedical Engineering College of Engineering Texas A&M University College Station TX 77843 USA; ^2^ Department of Cell Biology and Genetics College of Medicine Texas A&M University Bryan TX 77807‐3260 USA; ^3^ Interdisciplinary Program in Genetics and Genomics Texas A&M University College Station TX 77843 USA; ^4^ Department of Material Science and Engineering College of Engineering Texas A&M University College Station TX 77843 USA

**Keywords:** biomaterials, nanomaterials, regenerative medicine, RNA‐seq, stem cells

## Abstract

Minerals play a vital role, working synergistically with enzymes and other cofactors to regulate physiological functions including tissue healing and regeneration. The bioactive characteristics of mineral‐based nanomaterials can be harnessed to facilitate in situ tissue regeneration by attracting endogenous progenitor and stem cells and subsequently directing tissue‐specific differentiation. Here, cellular responses of human mesenchymal stem/stromal cells to traditional bioactive mineral‐based nanomaterials, such as hydroxyapatite, whitlockite, silicon‐dioxide, and the emerging synthetic 2D nanosilicates are investigated. Transcriptome sequencing is utilized to probe the cellular response and determine the significantly affected signaling pathways due to exposure to these inorganic nanomaterials. Transcriptome profiles of stem cells treated with nanosilicates reveals a stabilized skeletal progenitor state suggestive of endochondral differentiation. This observation is bolstered by enhanced deposition of matrix mineralization in nanosilicate treated stem cells compared to control or other treatments. Specifically, use of 2D nanosilicates directs osteogenic differentiation of stem cells via activation of bone morphogenetic proteins and hypoxia‐inducible factor 1‐alpha signaling pathway. This study provides  insight into impact of nanomaterials on cellular gene expression profile and predicts downstream effects of nanomaterial induction of endochondral differentiation.

## Graphical abstract

Transcriptomic profile reveals nanomaterials‐driven molecular mechanism to induce endochondral differentiation. This study compares and contrast various bioactive nanomaterials used in musculoskeletal tissue engineering.

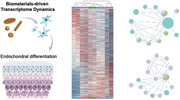

## 1 Introduction

Bioactive materials are pivotal in promoting in situ tissue regeneration, primarily by recruiting endogenous stem/progenitor cells to injury sites and guiding tissue‐specific differentiation, thereby facilitating tissue healing and regeneration.^[^ ^1^, ^2^ ^]^ These materials possess distinctive physicochemical properties that enable them to attract and influence endogenous stem cells through a combination of biochemical and biophysical cues.^[^ ^3^ ^]^ For instance, incorporating chemokines and growth factors into biomaterials can effectively draw endogenous cells and stimulate vascularization at damaged sites. Additionally, by emulating the extracellular matrix's stiffness and porosity, biomaterials can direct cell migration and proliferation. The strategic integration of cell homing peptides and the modulation of the local microenvironment via biomaterial degradation products further enhance cell recruitment. Given these capabilities, there is growing interest in the development of a new generation of bioactive or bioresponsive materials.^[^ ^4^, ^5^, ^6^ ^]^ These advanced materials aim to exploit the body's natural regenerative potential, representing a promising frontier in regenerative medicine and tissue engineering.

Among various biomaterials, inorganic nanomaterials provide a unique benefit for in situ tissue regeneration, due to their high surface‐to‐volume ratio. Both biophysical and biochemical characteristics of these nanomaterials such as size, shape, chemical composition, and surface chemistry plays a major role in directing stem cell fate.^[^ ^5^, ^7^ ^]^ For example, surface chemistry of nanoparticles dictate the formation of protein corona, while the size and shape of nanoparticles control the rate of cellular internalization. Once internalized, these inorganic nanomaterials can slowly degrade and release bioactive ions to direct cellular machinery and induce robust differentiation.^[^ ^8^ ^]^ Inorganic nanomaterials such as calcium phosphate, nano‐hydroxyapatite, and nano‐whitlockite have been extensively used in bone tissue regeneration strategies due to their structural and chemical similarities with mineralized tissue.^[^ ^9^ ^]^ Similarly, silicon‐based biomaterials such as bioactive glass, nanosilicates and nano‐silicon dioxide have shown bioactive characteristics and have been investigated in bone regeneration strategies.^[^ ^4^, ^8^, ^10^ ^]^ While these inorganic nanomaterials have been implicated for aiding in bone regeneration, little is known about their bioactive mechanism.

Here, we investigate the effect of commonly used bioactive nanomaterials on transcriptome dynamics of human mesenchymal stem stromal cells (hMSCs) and compare their osteoinductive ability to induce differentiation. We focus on four mineral nanomaterials: nanosilicates (nSi), nano‐hydroxyapatite (nHA), nano‐whitlockite (nWH), and nano‐silicon dioxide (nSiO_2_). Prior studies have utilized biased techniques, such as polymerase chain reaction (PCR) and microarrays, to study cellular responses which provide insufficient assessment of the affected biological processes.^[^ ^11^ ^]^ To overcome this shortcoming, we utilize whole transcriptome sequencing (RNA‐seq) to provide an un‐biased evaluation of the impact of these bioactive nanomaterials on transcriptome dynamics.^[^ ^12^ ^]^ We aim to use this technique to understand the effect of bioactive nanomaterials on hMSC cellular function and differentiation. Our approach delineates the unique effects of different inorganic nanomaterials on transcriptome dynamics of mesenchymal stem stromal cells during late‐stage osteogenic differentiation.

## 2 Results and Discussion

### 2.1 Physiochemical Characteristics of Inorganic Nanomaterials Induce Changes in Transcriptome Profile of hMSCs

#### 2.1.1 Physiochemical Characteristics of Inorganic Nanomaterials

**Figure 1:**
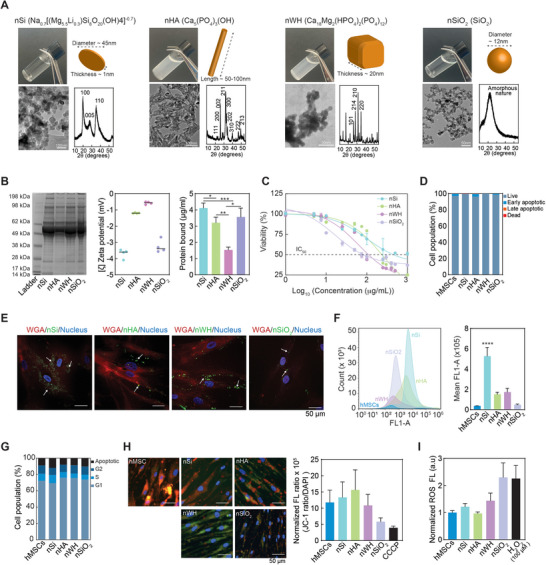
Physiochemical characteristic and cellular interaction of inorganic nanomaterials (nano‐hydroxyapatite (nHA), nano‐whitlockite (nWH), nanosilicates (nSi), and nano‐silicon dioxide (nSiO_2_)). A) Optical images show nanomaterial suspension in aqueous media (concentration 100 µg mL^−1^). Transmission electron microscopy (TEM) images provide size and shape of various nanomaterials. X‐ray diffraction (XRD) provide information about crystalline and amorphous characteristics as demonstrated by presence or absence of characteristics diffraction peaks. B) Interaction of bioactive inorganic nanomaterials with serum proteins are investigated using SDS PAGE. The surface charge after protein corona formation was determined using zeta potential measurements. Total protein content on the surface of bioactive nanomaterials was determined using Bradford assay (n = 3, ^*^p<0.05; ^**^p<0.01, ^****^p<0.0001). C) Effect of inorganic nanomaterial on hMSC viability is determined using cytotoxicity assay. Three technical replicates were used for each condition. Half‐maximal inhibitory concentration (IC_50_) is labeled at 50% viability. D) Effect of inorganic nanomaterials on cell apoptosis is determined using flow cytometry‐based PI‐Annexin V staining after 24 h (n = 3). E) Qualitative evaluation of uptake efficiency of inorganic nanomaterials using fluorescence microscopy. BM‐hMSCs were incubated with FITC‐BSA tagged nanomaterials for 24 h prior to imaging. F) Quantitative evaluation of inorganic nanomaterial uptake efficacy in hMSCs using flow cytometry after 24 h treatment with FITC‐BSA tagged nanomaterials (n = 3, ^****^p<0.0001). G) Effect of inorganic nanomaterials on cell cycle is determined after 24 h using flow cytometry (n = 3). H) JC‐1 mitochondrial membrane potential of BM‐hMSCs treated with 50 µg ml^−1^ of inorganic nanomaterials. I) ROS production in hMSCs treated with 50 µg ml^−1^ of inorganic nanomaterials for 24 h (n = 3).

The morphology and structure of nanomaterials affect cellular function in a variety of ways. Transmission Electron Microscopy (TEM) revealed the unique morphological features of different nanomaterials. nSi are 2D discs, 20–50 nm in diameter. nHA are elongated and rod‐like in shape with a typical length of 50–100 nm long and width of approximately 10 nm. nWH are observed to be rhombohedral, which closely correlates with the literature,^[^ ^13^ ^]^ and are typically 50 nm in size. nSiO_2_ have a spherical morphology and are 12 nm in size (**Figure** **1**A). X‐ray diffraction (XRD) validated the crystalline structure of these nanomaterials, showing the variable lattice structure as indicated by diffraction planes (Figure 1A). nSi have semi‐crystalline morphology, with characteristic diffraction planes. Crystallite size of nHA and nWH was calculated from the crystalline peaks using the Scherr formula for the highest intensity peaks. The typical values were calculated to be approximately 10 and 35 nm, respectively. nSiO_2_ showed an amorphous nature. The charged nature and aqueous stability of mineral nanomaterials was investigated using zeta potential measurements. The zeta‐potential (ζ) of nSi, nHA, nWH, and nSiO_2_ was approximately −22.4, −14.6, −13.6, and −26.4 mV, respectively (Figure S1A, Supporting Information). The zeta potential measurements indicate that these nanomaterials are stable in aqueous solution. Dynamic light scattering (DLS) was also performed to determine the nanomaterial average size distribution profile (Figure S1B, Supporting Information). We observed that all nanomaterials have average hydrodynamic diameters of less than 200 nm.

To determine the effect of various inorganic nanomaterials on aqueous stability under physiological conditions, the zeta potential of nSi, nHA, nWH and nSiO_2_ were evaluated in cell culture media with 10% fetal bovine serum (FBS). The molecular weight distribution of the adsorbed proteins was evaluated by Sodium Dodecyl Sulfate–Polyacrylamide Gel Electrophoresis (SDS‐PAGE). Analysis of the protein bands revealed that the nature of the nanomaterial does not influence the macroscopic profile of adsorbed proteins (Figure 1B). In cell culture media, the zeta potential of all inorganic nanomaterials exhibited a marked elevation, attributable to the adsorption of proteins onto the nanomaterial surfaces. nSi demonstrated markedly elevated protein adsorption relative to other nanomaterials. This might be attributed to the high surface area and charged characteristics of the nSi compared to other nanomaterials.

To assess the biological response of these nanomaterials, we determine their cellular compatibility by monitoring metabolic activity, morphology and cell cycle. The half‐maximum inhibitory concentration (IC_50_) of different nanomaterials was determined as >1000 µg mL^−1^ for nSi, 300 µg mL^−1^ for nHA, and 100 µg mL^−1^ for nWH and 75 µg mL^−1^ for nSiO_2_ (Figure 1C). These results were similar to previous reports.^[^ ^8^, ^14^ ^]^ To avoid stress induced by nanomaterial uptake, we used a concentration of 50 µg mL^−1^, which is lower than IC_50_ of all the nanoparticles. To further verify that the selected nanomaterial concentration of 50 µg mL^−1^ does not influence apoptosis, we performed using flow cytometry‐based PI‐Annexin V staining apoptosis assay (24 h). As expected, no significant change in cell shape or spreading was observed (Figure S1D, Supporting Information). No significant changes in apoptotic cell population were observed between the various treatment groups as well (Figure 1D). Based on our data, we selected nanomaterial concentrations of 50 µg mL^−1^ for all future analyses.

Intracellular uptake using fluorescence microscopy and subsequent quantification using flow cytometry confirmed that nSi was internalized by hMSCs at a higher rate (≈ threefold increase) than other inorganic nanomaterials (Figure 1E,F) Similarly, no significant change in cell cycle (G1/S/G2 phases) was observed (Figure 1G). However, analysis of mitochondrial health using the JC‐1 assay, which monitors mitochondrial membrane depolarization revealed nWH and nSiO_2_ cause some degree of depolarization of mitochondrial membrane, although not statistically significant as compared to nSi and nHA (Figure 1H). These effects were also subsequently observed in reactive oxygen species (ROS) assay, wherein in nSiO_2_ showed slightly higher ROS production than other groups (although not statistically significant), indicating possible cellular stress (Figure 1I).

#### 2.1.2 Nanomaterials Induce Changes in hMSCs Transcriptome Dynamics

The inorganic materials nSi, nHA, nWH, and nSiO_2_ have previously been identified as bioactive nanomaterials, however to our knowledge their bioactivity on stem cell differentiation has not been compared before. To evaluate the cellular response to nanomaterials, we performed whole‐transcriptome sequencing (RNA‐seq) of cells treated with different nanomaterials (nSi, nHA, nWH, and nSiO_2_) (see Experimental Methods). Cells were cultured in osteoconductive media (OC) containing β‐glycerophosphate and L‐ascorbic acid without any osteoinductive additives such as dexamethasone or recombinant bone morphogenic protein 2 (rBMP‐2) to evaluate relative osteoinductivity of different nanomaterials. The experiment was performed in two technical replicates (n = 2). The sequenced reads were aligned to the human genome (hg38) using an RNA‐seq aligner^[^ ^15^ ^]^ and gene expression levels were determined by calculating fragments per kilobase of transcript per million (FPKM). Generalized linear models (GLMs) as described in DESeq2^[^ ^16^ ^]^ were used to obtain differentially expressed genes between different conditions (DEGs; *p‐adj* < 0.05) (Dataset S1, Supporting Information).

**Figure 2:**
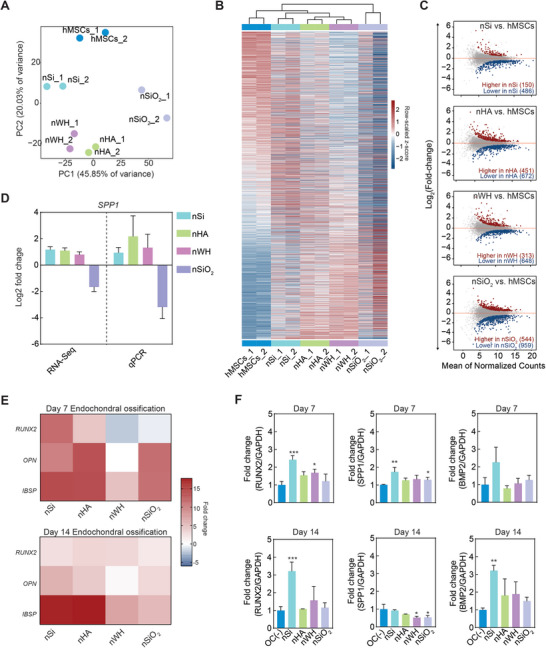
Effect of nanomaterials (nSi, nHA, nWH, and nSiO_2_) on hMSCs transcriptome and their influence on endochondral ossification. A) Principal component analysis (PCA) of hMSC samples treated with nanomaterials (nSi, nHA, nWH, and nSiO_2_) based on mRNA expression obtained from RNA‐seq. Cells without nanomaterials is used as control (hMSCs). The PCA analysis was done on the mRNA expression (Log_2_FPKM) of 20% most variable genes (determined by median absolute deviation) across all samples (n = 2,373). Two technical replicates were used for each condition. B) Hierarchical clustering of hMSC samples treated with (nSi, nHA, nWH, and nSiO_2_) and without nanomaterials (hMSCs) based on mRNA expression obtained from RNA‐seq. The heatmap shows the differentially expressed genes (Log_2_FPKM of DEGs, *p‐adjust* < 0.05) across all nanomaterial treatment groups compared to control hMSC samples (n = 2,679). Effects of nHA and nWH treatments are most similar, perhaps due to their similar chemical composition C) Difference in gene expression (Log_2_(*Fold change*)) between hMSC samples treated with nanomaterials and control (hMSCs). A generalized linear model (GLM) was used to identify significant events. Genes with significantly high expression in hMSCs treated with nanomaterials are shown in red (*p‐adjust* < 0.05) and genes with significantly low expression (*p‐adjust* < 0.05) are shown in blue. Grey denotes genes that are expressed in the samples being compared but do not exhibit significantly different expression. D) Differential gene expression from RNA‐seq was validated using qRT‐PCR, indicating similar trend (n = 3, ^*^p<0.05; ^**^p<0.01, ^****^p<0.0001). E) qRT‐PCR against markers of endochondral ossification‐runt related transcription factor‐2 (*RUNX2*), osteopontin (*OPN*) and bone sialoprotein II (*IBSP*) at Day 7 and Day 14 post treatment with inorganic nanomaterials. F) Protein level validation of endochondral ossification markers (RUNX2, SPP1/OPN, BMP2) at Day 7 and Day 14 using enzyme linked immunosorbent assays (ELISA) (n = 3, ^*^p<0.05; ^**^p<0.01, ^***^p<0.001).

Our results showed that these nanomaterials have distinct effects on the transcriptome profile of hMSCs (**Figure** **2**A,B). Interestingly, principal component analysis (PCA) of 20% most variable genes from the cohort as well as hierarchal clustering of DEGs indicated higher similarity between nHA and nWH treated cells in comparison to other treatment groups. We speculate that this similarity in cellular response may be driven by their similar chemical compositions. PCA and hierarchical clustering of the samples also validates the limited variation within the control and treatment replicates. Compared to untreated cells, cells treated with nSi, nHA, nWH, and nSiO_2_ significantly changed the expression of 636, 1,123, 961, and 1,503 genes respectively (DEGs, *p‐adj* < 0.05) (Figure 2C). Interestingly, only 68 genes were common between all four groups of DEGs (Figure S2B, Supporting Information).

### 2.2 Bioactive Nanomaterials Promote Endochondral Differentiation

Endochondral differentiation is a multi‐stage process which forms most of the long bones in the body.^[^ ^17^, ^18^ ^]^ Briefly, the early stages involve the formation of a cartilaginous template from mesenchymal condensation. The endogenous progenitor or stem cells in this stage proliferate and differentiate into chondrocytes. A dense layer of perichondrium connective tissue then forms around the periphery of this cartilage template which triggers matrix metalloprotease 13 (MMP13) production resulting in chondrocyte hypertrophy. These hypertrophic chondrocytes then express markers such as collagen type X (COLX), bone morphogenetic protein 2 (BMP2), and the transcription factors, *RUNX2* and *SPP1* further promoting endochondral differentiation.^[^ ^17^ ^]^ To understand the bioactive effect of nanomaterials on stem cells, expression levels of DEGs involved in chondro‐, osteo‐, and endochondral differentiation were investigated. Genes associated with chondrogenesis such as *COMP* and *ACAN* were shown to be downregulated or not regulated at all by nanomaterials at day 21. The *SPP1* gene codes for the protein osteopontin (OPN), which is a key marker for ossification and is highly expressed in osteoblasts. The change in mRNA expression levels of *SPP1* via RNA‐seq was further validated using qRT‐PCR (Figure 2D). Additionally, some genes associated with osteogenesis such as *ALP* or *COL1A1* were downregulated by nanomaterials.

**Figure 3:**
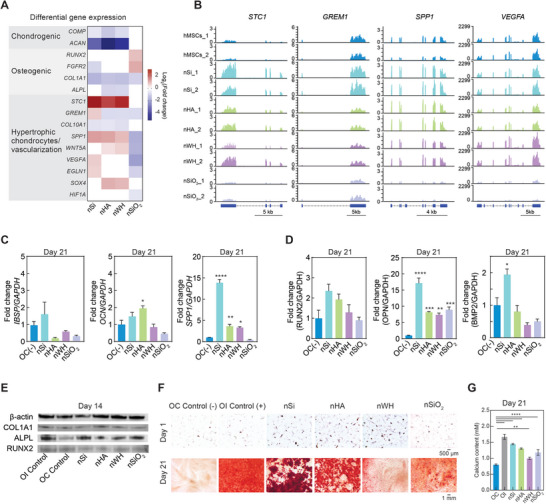
Effect of nanomaterials (nSi, nHA, nWH, and nSiO_2_) on endochondral differentiation of hMSCs. A) DEGs (*p‐adjust <* 0.05) associated with chondrogenesis, osteogenesis, and hypertrophic chondrocyte‐skeletal progenitors showing the effect of nano gene expression (Log_2_(*Fold change*)) caused by nanomaterial treatments (green: up‐regulated, red: down‐regulated). Hypertrophic chondrocyte‐skeletal progenitor markers are uniquely activated by nSi compared to other mineral nanomaterials. B) RNA‐seq tracks showing normalized mRNA expression (aligned reads normalized by total library–size—transcript per million (TPM)) at the genomic locus of vascular endothelial growth factor A (*VEGFA*), gremlin 1 (*GREM1*), secreted phosphoprotein 1 (*SPP1*), and stanniocalcin 1 (*STC1*) for hMSCs and nanomaterials treated hMSCs. C) Relative mRNA expression levels of endochondral ossification genes (*IBSP*, *OCN* and *SPP1*) at Day 21 post treatment with various bioactive inorganic nanomaterials determined by qRT‐PCR (n = 3, ^*^p<0.05; ^**^p<0.01, ^****^p<0.0001). D) Relative expression of endochondral markers at Day 21 determined by enzyme linked immunosorbent assay (n = 3, ^*^p<0.05; ^**^p<0.01, ^***^p<0.01, ^****^p<0.0001). E) Effect of nanomaterials treatment on production of osteo‐specific protei–s—alkaline phosphatase (ALP), collagen type I alpha 1 (COL1A1), and Runt‐related transcription factor 2 (RUNX2) is determined using western blot. Amount of protein is normalization by house‐keeping protein (β‐actin) for quantitative assessment. F) Qualitative assessment of mineralized matrix production due to nanomaterial treatment is performed by staining calcium deposits using Alizarin Red S (red). hMSCs cultured in absence and presence of osteoinductive agents (dexamethasone) is used as negative and positive controls. G) Amount of calcium quantified from the mineralized matrix obtained by quantifying the amount of Alizarin Red S in samples treated with different conditions for 21 days. Statistical comparison between samples is performed using one‐way ANOVA and Dunnett's multiple comparison test, with a single pooled variance (n = 3, ^****^ *p* < 0.0001, ^**^ *p* <0.01).

Endochondral markers such as *IBSP*, *OCN*, *OPN*, *RUNX2* and *BMP2* are crucial markers for successful conversion of hypertrophic chondrocytes into mineralized bone and were upregulated by nanomaterials which was also confirmed both at mRNA and protein levels in two different lots of hMSCs (Figure 2E,F). Specifically, nSi and nHA showed high mRNA levels of *RUNX2*, *OPN* and *IBSP* at Day 7 and Day 14 as compared to nWH and nSiO_2_ treatments. nSi in particular showed high upregulation for each of the markers (*RUNX2*:9.8‐fold increase, *OPN*:8.37‐fold increase and *IBSP* ≈ 12‐fold increase). A similar trend was also observed at the protein level where nSi treatment resulted in two to threefold increase in the production of these markers. Studies have shown that during endochondral ossification, chondrocytes condense and become hypertrophic in a hypoxic environment, mineralize, and enhance the expression of vascular endothelial growth factor (VEGF) to promote new blood vessel invasion for bone formation.^[^ ^19^ ^]^ Both hypertrophic chondrocytes and osteoblasts have been shown to produce *VEGFA*,^[^ ^20^ ^]^ which was also found to be significantly (*p‐adj* < 0.05) upregulated with nSi treatment in our study (**Figure** **3**A). Further, gremlin 1 (*GREM1)* is a well‐established marker to identify skeletal progenitor‐hypertrophic chondrocytes.^[^ ^21^ ^]^ We determined that nSi treatment significantly increases the expression of *GREM1*, unlike nHA, nWH, and nSiO_2_ (Figure 3B). Additionally, nSi, nHA, and nWH upregulated secreted phosphoprotein 1 (*SPP1*) (also known as osteopontin *OPN*) which is highly expressed in terminal hypertrophic chondrocytes.^[^ ^22^ ^]^ nSi, nHA, and nWH also upregulated the expression of stanniocalcin 1 (*STC1*) which has previously been identified as a marker of mineralization during osteogenesis.^[^ ^23^, ^24^ ^]^ Overall, nSi led to increased expression in each key endochondral marker unlike other nanomaterials which only affected a subset of these genes. Upregulation of endochondral, and downregulation of osteo‐ and chondro‐genes at day 21 was used to determine skeletal progenitor‐hypertrophic chondrocyte behavior. The mRNA levels of *IBSP* and *SPP1* were found to be the highest in nSi treated hMSCs at 1.6‐fold increase and 14‐fold increase respectively (Figure 3C). This was again confirmed at protein level wherein nSi treatment resulted in 17‐fold increase in SPP1, 1.9‐fold increase in BMP2 and 2.3‐fold increase in RUNX2 levels (Figure 3D). Further, this analysis indicated stronger bioactivity of nSi to direct endochondral differentiation compared to other nanomaterials at transcriptomic level.

Subsequently, western blot analysis was performed to evaluate the expression of osteo‐specific proteins at day 14 (Figure 3E). Of note, nSi treatment resulted in an approximately fourfold increase in alkaline phosphatase (ALP) expression compared to the control (hMSCs only). Type I collagen alpha 1 (COL1A1) expression was slightly higher in nHA samples than controls at day 14. While alkaline phosphatase (ALP) expression was higher for nSi treated hMSCs and runt‐related transcription factor 2 (RUNX2) expression was highest in nWH treated hMSCs. Expression of ALP is encouraging to observe in nanomaterial treated samples as this protein is associated with osteogenesis among other biological processes. Further, ALP expression was similar in nSi samples and positive control (osteoinductive media (OI)). OI media containing β‐glycerophosphate, L‐ascorbic acid and osteoinductive agent‐dexamethasone. However, limited expression of osteo‐related proteins such as RUNX2 and COL1A1 at the day 14 timepoint despite the addition of these known osteo‐related nanomaterials may suggest another mechanism aside from intramembranous ossification is occurring to result in the deposition of mineralized matrix.

To understand how bioactive nanomaterials promote osteogenesis, we performed late‐stage osteogenesis assays (14‐ and 21‐day time‐points). Nanomaterials were replenished along with media every 3–4 days. After 14 days, an ALP assay was performed, as a preliminary assessment for the start of osteogenesis, and revealed no significant changes in expression between each nanomaterial treatment (Figure S4, Supporting Information). Day 21 Alizarin Red S stain showed all nanomaterials and the osteoinductive (OI) control had significantly more mineralized matrix deposition than the negative, osteoconductive control (OI, nSi, nHA, and nSiO_2_^****^ *p* < 0.0001; nWH ^**^ *p* <0.01) (Figure 3F). Day 1 Alizarin Red staining confirmed the addition of calcium phosphate nanomaterials nHA and nWH did not result in a significant increase in staining (Figure S4, Supporting Information). Therefore, day 21 staining was not disproportionally skewed by the addition of nHA and nWH. Ultimately, quantification of mineralized matrix deposition suggests that nSi and perhaps nHA are strong candidates to promote tissue ossification compared to nWH and nSiO_2_ (Figure 3G).

### 2.3 Bioactive Nanomaterial Trigger Multiple Signaling Pathways to Induce Endochondral Ossification

**Figure 4:**
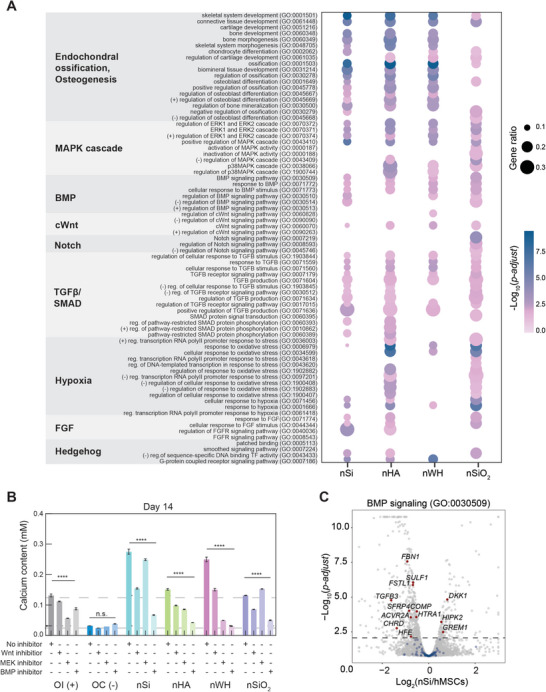
Molecular mechanism triggered by bioactive nanomaterials (nSi, nHA, nWH, and nSiO_2_) to regulate endochondral ossification. A) Bubble plot highlighting the significantly affected biological process (*p‐adj <* 0.05) related to endochondral ossification, osteogenesis, mitogen‐activated protein kinase (MAPK), bone morphogenetic protein (BMP), and canonical Wingless‐related integration site (cWnt), notch, transforming growth factor beta/SMAD (TGF β/SMAD), hypoxia, fibroblast growth factors (FGF), and hedgehog. Circle size correlates to the gene ratio (DEGs associated with a GO term divided by the total number of expressed genes mapped to the GO term). Color intensity is associated with −Log_10_(*p‐adj*). B) Role of different signaling pathways to induce stem cell differentiation is identified by determining amount of mineralized matrix in presence or absence of nanomaterials and/or pathway‐specific–inhibito–s—cWnt (10 mm), MAPK (5 µm), and BMP signaling (5 µm). The amount of calcium in the extracellular matrix (ECM) is determined using Alizarin Red S quantification on Day 14. Statistical comparison between samples is performed using one‐way ANOVA and Dunnett's multiple comparison test, with a single pooled variance (n = 3). hMSCs cultured in absence and presence of osteoinductive agents (dexamethasone) is used as negative and positive controls. C) A volcano plot showing DEGs (*p‐adj* < 0.05) for BMP signaling (GO:00 30509). Grey: all of the expressed genes, blue: genes associated with the GO term with no significant change in expression, red: genes associated with the GO term that show significant difference in expression due to nanosilicate treatment.

We utilized the list of DEGs to determine the biological processes affected by nSi, nHA, nWH, and nSiO_2_ in promoting endochondral ossification. The key biological processes were identified via gene ontology (GO) enrichment analysis (see Experimental Methods). Our results shows the significantly affected GO biological processes associated with osteogenesis and endochondral ossification, specifically, the ones related to mitogen‐activated protein kinase (MAPK), bone morphogenetic protein (BMP), and canonical Wingless‐related integration site (cWnt), notch, transforming growth factor beta/SMAD (TGFβ/SMAD), hypoxia (hypoxia‐inducible factor (HIF)), fibroblast growth factors (FGF), and hedgehog (sonic hedgehog (Shh), and Indian hedgehog (Ihh)) (**Figure** **4**A; Datasets S2–S5, Supporting Information). These biological process have been identified as key signaling pathways that regulate endochondral ossification and development.^[^ ^25^ ^]^ (Figure 4B).

To elucidate the impact of nanomaterials on osteogenic differentiation, we conducted inhibition studies targeting the cWnt, MAPK, and BMP signaling pathways, which were identified as significant through GO enrichment analysis. These studies aimed to assess the subsequent effects on matrix mineralization at day 14 (Figure 4B). We observed that inhibition of the BMP pathway markedly reduced matrix mineralization across all nanomaterial‐treated human mesenchymal stem cells (hMSCs) (nSi, nHA, nWH, and nSiO2; ^****^p < 0.0001). Notably, the BMP inhibitor treatment in nSi‐treated groups led to a sixfold decrease in calcium content compared to controls, underscoring the critical role of the BMP pathway in osteogenic differentiation induced by nanomaterials. Additionally, Wnt pathway inhibition in nSi‐treated cells resulted in a 2.3‐fold reduction in calcium content, highlighting the interconnectedness of these signaling pathways in osteogenic processes. Previous studies have shown that these osteogenic pathways are intertwined, as BMP activates cWnt and/or MAPK during osteogenic differentiation.^[^ ^26^ ^]^ Therefore, it was unsurprising that addition of cWnt and MAPK inhibitors showed some negative effect on the production of mineralized matrix in all nanomaterial treated samples (nSi, nHA, nWH, and nSiO_2_; ^****^ *p* < 0.0001). These results differed from the positive control group (OI) which showed the largest decrease with addition of MAPK inhibitor though all inhibitors caused a significant decrease (OI; ^****^ *p* < 0.0001). There was no significant difference in mineralized matrix in the negative control treated with the inhibitors. These results suggest that BMP signaling plays a large role in mediating the production of mineralized matrix in nanomaterial treated hMSCs, which is supported by GO enrichment analysis.

BMP signaling has also been associated with endochondral differentiation, and works in tandem with other spatiotemporal activating factors to control cartilaginous template formation and subsequent ossification. The key pathways identified in Figure 4A all interface with BMP pathway, whether by activating or being activated by this signaling cascade.^[^ ^26^, ^27^ ^]^ Based on our BMP inhibition investigation, this signaling cascade may play a role in regulating other pathways associated with endochondral differentiation. The gene ontology term BMP signaling (GO:00 30509) shows regulation of the skeletal progenitor‐hypertrophic chondrocyte marker *GREM1*, which is upregulated in nSi samples (Figure 4C; Figure S3B, Supporting Information). Going further, we employ additional analysis techniques to elucidate unique, day 21 endochondral pathway activation by nanomaterials.

### 2.4 Gene Set Enrichment Analysis Identify Role of Nanomaterials in Matrix Remodeling

**Figure 5:**
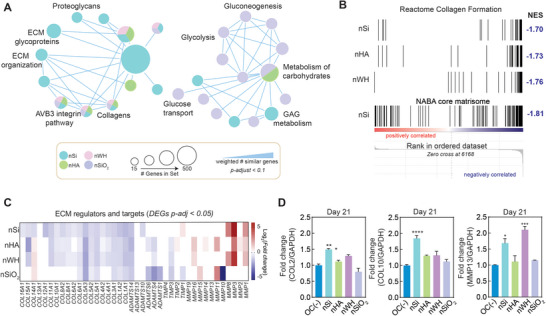
Gene set enrichment analysis (GSEA) yields insights about the effect of nanomaterials on matrix remodeling. A) GSEA graph displaying interconnected gene sets of hMSC treated with different nanomaterials. The graph was generated in Cytoscape with Enrichment Map using enriched gene sets *p‐adj* <0.1 and an edge similarity coefficient > 0.375. The effect of nanomaterials on extracellular matrix organization, glycoproteins, and proteoglycans is shown. B) GSEA enrichment results show normalized enrichment scores (NES) for *Reactome* gene set *Collagen Formation* and *NABA core matrisome*. The vertical black lines (“bar code”) represents the projection onto the ranked gene list of individual genes of the gene set. The horizontal bar in graded color from red (left) to blue (right) represents the gene list ranked from upregulated on the left to downregulated the right. C) Heat map of DEGs (*p‐adj <*0.05), Log_2_(*fold change*) expression of genes associated with matrix remodeling including matrix metalloproteinases (MMPs), tissue inhibitors of metalloproteifferentiaPs), a disintegrin and metalloproteinase with thrombospondin motifs (ADAMTSs), and collagens identified through RNA‐seq (red: up‐regulated, blue: down‐regulated). MMP transcripts are upregulated by nSi, nHA, and nWH, including MMPs. Other matrix remodeling enzymes such as ADAMTSs and TIMPs are downregulated by nanomaterials. Collagens are mostly downregulated, except *COL14A1* which was upregulated by nHA, nWH, and nSiO_2_. D) Relative protein expression of collagen type 2 (COL2A1), collagen type X (COL10) and matrix metalloprotease 13 (MMP13) in hMSCs and inorganic nanomaterial treated hMSCs determined by ELISA (n = 3, ^*^p<0.05; ^**^p<0.01, ^***^p<0.01, ^****^p<0.0001).

GO enrichment analysis alone may miss key pathways perturbed by nanomaterials due to DEG cutoffs used in analysis (in our case, *p‐adj* < 0.05). Therefore, we also performed gene set enrichment analysis (GSEA) to evaluate the dynamics of all expressed genes, taking into account all statistically significant and insignificant perturbations through a pre‐ranked list.^[^ ^28^ ^]^ We used GSEA to determine whether a priori, curated gene sets from the Molecular Signature Database (MsigDB: v7.2) exhibited statistically significant concordant differences in treated versus untreated hMSCs. GSEA determines the enrichment of these curated gene sets in our own pre‐ranked gene list (see Experimental Methods). Gene sets with a false discovery rate (FDR) adjusted (*p‐adj) <* 0.1 were considered enriched in our own pre‐ranked list. GSEA results were imported into Cytoscape to make a network linking enriched gene sets based on gene overlap using the Enrichment Map function (**Figure** **5**A). The node generation cut‐off for gene sets was set to a *p‐adj* value of 0.1, and an edge similarity coefficient of > 0.375 to describe gene similarity between sets. The Enrichment Map suggests that nSi significantly regulates gene sets associated with extracellular matrix including glycoproteins, proteoglycan and collagens. All nanomaterials have a strong effect on *AVB3 Integrin Pathway*. Regulation of these gene sets by nanomaterials indicate a strong effect on extracellular matrix interactions and organization. In addition, significantly regulated genes were involved in the *Naba* gene set *Collagens*, and the *Reactome* gene set *Collagen Formation*. Specifically, nSi was shown to regulate the *Reactome* gene set *Glycosaminoglycan Metabolism*. nSi and nWH controlled the *Kegg* gene sets *ECM Receptor Interaction* and *Focal Adhesion*.

GSEA takes into account the direction of gene expression (whether a gene undergoes upregulation or downregulation) due to treatment which is not accounted for in GO enrichment analysis. GSEA provides an Enrichment Score (ES), which reflects the degree to which a gene set is overrepresented at the top or bottom of a ranked list of genes. Normalized enrichment scores (NES), which accounts for differences in gene set sizes within different treatment group, indicated that treatment with nSi, nHA and nWH resulted in negative NES for the *Reactome* gene set *Collagen Formation* (Figure 5B).

Regulation of the matrisome and other extracellular matrix organization suggests that matrix remodeling is occurring. Matrix remodeling is expected during endochondral ossification, and several matrix metalloproteinases (*MMPs*) associated with this process are upregulated by nSi, nHA, or nWH (Figure 5C). nSi and nWH upregulate *MMPs* 1 and 8 which are considered “collagenases”.^[^ ^29^ ^]^ *MMP1* and *MMP3* (a stromelysin upregulated by nSi, nHA and nWH) are known to be expressed during endochondral ossification.^[^ ^30^ ^]^ Further, nSi and nHA upregulated tissue inhibitors of matrix metalloproteinases (*TIMP*)1 and 2, respectively, which are often found in hypertrophic chondrocytes.^[^ ^30^, ^31^ ^]^ Glycoproteins such as thrombospondin 1 (*THBS1)* (chondroprotective and prevents ossification)^[^ ^32^ ^]^ are downregulated by nSi, further support the ability of nSi to induce matrix mineralization. nSiO_2_ treatment resulted in less regulation of collagen expression and did not upregulate key *MMP*s as with other nanomaterials. nSiO_2_ treatment regulated gene sets associated with the extracellular matrix. However, DEG expression, GSEA and GO enrichment analysis suggest that nSiO_2_ do not regulate extracellular matrix organization or endochondral differentiation processes to the same extent as nSi, nHA, or nWH. These observations were also confirmed by evaluating relative expression of COL2, COL10 and MMP13 proteins, which are key markers of endochondral ossification and matrix remodeling in the context of bone formation. (Figure 5D).

### 2.5 Nanosilicates Control SOX4 Targets via Activation of HIF Pathway

**Figure 6:**
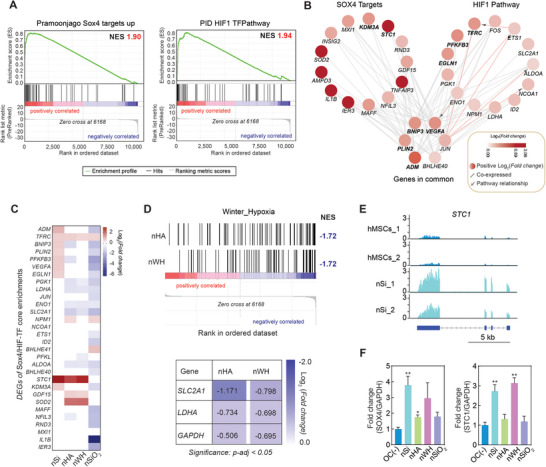
Nanosilicates activate Sox4 and HIF‐1α signaling pathway to induce endochondral differentiation. A) GSEA shows positive normalized enrichment scores (NES) for *Pramoonjago:Sox4 Targets* and *PID: HIF1 TFPathway*. The curve in green corresponds to the calculation of the enrichment score (ES). The vertical black lines (“bar code”) represent the projection onto the ranked gene list of the individual genes of the gene set. The horizontal bar in graded color from red (left) to blue (right) represents the gene list ranked from positive correlated on the left to negative correlated on the right. B) Network analysis generated in Cytoscape using GeneMANIAdepict gene set overlap, genetic co‐expression, and pathway interaction between Pramoonjago:Sox4 Targets and PID:HIF1 TFPathway which are upregulated by nSi. Only core enrichments to each gene set were included, and the significant DEGs(p‐adj〈0.05) are highlighted in bold black text (KFM3A, STC1, EGLN1, PFKFB3,TFRC,BNIP3,PLIN2,and VEGFA). C) Heatmap of DEG (p‐adj〈0.05) associated with core enrichments of Pramoonjago:Sox4 Targets and PID: HIF1 TFPathway identified through GSEA analysis of nSi treated hMSCs. Log2(Fold change) expression (White: no significant change; Red; up‐regulated, Blue:down regulated). D) Gene set enrichment plots for *Winter Hypoxia* downregulated by nHA and nWH, respectively. Genes within *Winter Hypoxia* gene sets that associated with glycolysis are downregulated in samples treated with nHA and nWH, such as solute carrier family 2 member 1 (*SLC2A1*) and lactate dehydrogenase A (*LDHA*) (*p‐adj* < 0.05). E) RNA‐seq tracks showing normalized mRNA expression (aligned reads normalized by total library–size—transcript per million (TPM)) at the genomic locus of stanniocalcin 1 (*STC1*) for hMSCs and nSi treated hMSCs. F) Relative protein expression of SOX4 and STC1 in hMSCs and nanomaterial treated hMSCs at Day 21 determined by ELISA (n = 3, ^*^p<0.05; ^**^p<0.01).

GSEA results were further analyzed for a priori gene sets that could help in identifying processes triggered by nSi which facilitated deposition of mineralized matrix. We found that the genes present in *Pramoonjago: Sox4 Targets UP* and *PID: HIF TFPathway* (**Figure** **6**A) gene set were enriched at the top of the list, indicating their tendency to exhibit increased expression in response to nSi treatment. Previous literature has suggested that SRY‐Box Transcription Factor 4 (Sox4) promoted cellular proliferation and chondrogenesis.^[^ ^33^ ^]^ Hypoxia signaling pathway, which is predominantly governed by hypoxia‐inducible factor 1‐alpha (HIF‐1α) has shown to activate SOX4^[^ ^34^ ^]^ and is known regulator of BMP2‐induced endochondral ossification.^[^ ^35^ ^]^ Thus, both SOX4 and hypoxia signaling pathway can contribute to endochondral ossification.^[^ ^36^ ^]^ Based on our GSEA data, hypoxia signaling could be an important amplifier of ossification due to nSi treatment.

We validated our hypothesis by evaluating genes in both *Pramoonjago: Sox4 Targets UP* and *PID: HIF TFPathway*. Cytoscape was utilized to evaluate gene co‐expression and pathway interactions between *Pramoonjago: Sox4 Targets UP* and *PID: HIF TFPathway* (Figure 6B). Intriguingly, nSi upregulates genes in both these gene sets, unlike other nanomaterials (Figure 6C). As GSEA takes into account all DEGs, the network includes some genes that did not make the significance cut off (*p‐adj <* 0.05) but were core enrichments of the GSEA. Core enrichments are genes that contribute most to the GSEA enrichment result. *VEGFA* is an overlapping gene between *Sox4 Targets* and *HIF TFPathway*, and was previously shown to co‐express alongside the mineralization gene *STC1* in induced bone MSCs.^[^ ^37^ ^]^ *STC1* has previously been identified as a mineralization stimulant during development,^[^ ^23^ ^]^ has been shown to interact with calcitonin G‐protein coupled receptors necessary for osteogenesis,^[^ ^24^ ^]^ and is also associated with angiogenesis.^[^ ^38^ ^]^ Positive enrichment of genes such as *VEGFA* and *STC1* in GSEA highlights a potential mechanism by which hypertrophic chondrocyte expression or matrix mineralization could occur.

To further strengthen this observation, we also evaluated effect of other nanomaterials on these signaling pathways. Both nHA and nWH showed significant (*p‐adj* < 0.01) downregulation of gene sets related to hypoxia, specifically the gene set *Winter Hypoxia* (Figure 6D). Key genes such as solute carrier family 2 member 1 (*SLC2A1*) and lactate dehydrogenase A (*LDHA*) within this gene set were also downregulated, which shown that HIF‐1α signaling pathways is suppressed.^[^ ^39^ ^]^ While no term related to hypoxia was observed due to nSiO_2_ treatment, this offers a possible explanation for the mechanism by which nSi induces enhanced endochondral ossification at day 21 compared to other nanomaterials by upregulating the levels of both SOX4 and STC1 simultaneously (Figure 6F).

## 3 Challenges and Future Directions

Inorganic nanoparticles, including nanosilicates, nanohydroxyapatite, nanowhitlockite, and silica nanoparticles, are extensively employed in osteointegrative bone tissue regeneration due to their bioactive properties. Despite their widespread use, the impact of these bioactive nanomaterials on transcriptome dynamics has not been fully explored.^[^ ^1^, ^40^, ^41^ ^]^ While the majority of existing studies have concentrated on the effects of these nanomaterials on stem cell differentiation, relying predominantly on PCR or microarray technology, this method offers limited insight, focusing primarily on context‐specific investigations without a comprehensive evaluation of the affected biological processes.^[^ ^40^, ^42^, ^43^ ^]^ In contrast, high‐throughput bulk RNA sequencing addresses this limitation by enabling the analysis of multiple interacting signaling pathways activated by nanomaterial treatment.^[^ ^5^, ^6^, ^8^, ^40^ ^]^ This approach provides a comprehensive understanding of nanomaterial‐cell interactions, facilitating the prediction of downstream effects, such as the induction of endochondral differentiation by nanomaterials.^[^ ^40^ ^]^ Consequently, this method promotes the improved design of regenerative nanomaterials for bone tissue engineering by offering a holistic insight into the mechanisms of action and potential therapeutic applications of these bioactive compounds.

While our current study introduces bulk RNA‐Seq as a potentially unbiased method for investigating nanomaterial‐cell interactions, paving the way for incorporation of more next‐generation sequencing (NGS) technologies in this field, we must acknowledge several limitations within our study design. First, the necessity for knockdown studies is needed to validate specific pathways activated by inorganic nanomaterials, in conjunction with small molecule inhibitor study presented here. Small molecule inhibitors, while useful, often produce off‐target effects and exhibit a degree of non‐specificity.^[^ ^44^ ^]^ These inhibitors target the bioactivity of proteins without leading to their complete degradation, potentially allowing the protein to remain functionally active and participate in complex formations. This scenario can result in diminished activity and signaling crosstalk. Additionally, the efficacy of small molecule inhibitors is contingent upon precise dosing schedules, beyond which their effects may diminish. Therefore, verifying the outcomes of small molecule inhibition through knockdown studies is essential for a more accurate interpretation of results.

Second, our study highlights the confounding influence of both the chemical composition and physical structure of nanomaterials on transcriptome dynamics. Other studies have shown that nanoparticle size significantly impacts osteogenic differentiation outcomes in a dose‐dependent manner, with smaller particles (50 and 100 nm) demonstrating enhanced osteogenic differentiation and reduced cytotoxicity in hMSCs compared to larger particles (150 nm).^[^ ^45^ ^]^ Similarly, the shape of hydroxyapatite (HA) particles significantly affects cell behavior; spherical HA particles, for example, are more efficacious in promoting cell proliferation and osteogenic differentiation.^[^ ^46^ ^]^ The necessity for in vivo studies to confirm the translational efficacy of proposed nanomaterials also represents a crucial aspect of experimental design for future research endeavors.^[^ ^47^ ^]^

Although bulk RNA‐seq has shed light on the impact of various nanomaterials on the transcriptomic profiles of cells, assessing this effect at the single‐cell level is imperative. Single‐cell RNA sequencing (scRNA‐seq) offers the potential for profound insights into the degree of differentiation and the biological processes influenced by nanomaterial exposure. Specifically, scRNA‐seq enables the analysis of thousands of individual cells, thus delineating cellular heterogeneity with single‐cell resolution. In the future, the application of single‐cell analysis is anticipated to uncover novel insights regarding the effects of nanomaterial treatments on cellular diversity, characterize the array of cell types within the local microenvironment, and elucidate signaling interactions among different cell populations. This approach promises to enhance our understanding of the nuanced effects of nanomaterials on cellular behavior and the intricate dynamics of tissue regeneration and repair.

## 4 Conclusion

We investigate cellular responses of human mesenchymal stem stromal cells (hMSCs) to traditional bioactive mineral‐based nanomaterials, such as hydroxyapatite (nHA), whitlockite (nWH), silicon‐dioxide (nSiO_2_), and the emerging synthetic 2D nanosilicates (nSi). We utilized transcriptome sequencing (RNA‐seq) to probe the cellular response and determine the significantly affected signaling pathways due to exposure to these inorganic nanomaterials. RNA‐seq revealed that nSi induce a stabilized skeletal progenitor state suggestive of endochondral differentiation. This observation is bolstered by enhanced deposition of matrix mineralization in nSi treated stem cells compared to control or other treatments.

Gene set enrichment analysis (GSEA) show activation of hypoxia‐inducible factor (HIF) signaling pathway due to nSi treatment which was absent in nHA, nWH, and nSiO_2_. HIF signaling is activated by BMP signaling, as supported by gene ontology (GO) enrichment analysis. Pathway inhibitor studies further support the role of nSi in triggering BMP signaling, which further activate HIF pathway. Overall, this study provides a holistic transcriptomic insight into nanomaterial‐cell interactions and predicts downstream effects of nanomaterial induction of endochondral differentiation.

## 5 Experimental Section

### Materials

Laponite XLG, a synthetic clay nanosilicate (nSi) with the formula Na^+^_0.7_[(Mg_5.5_Li_0.3_Si_8_O_20_(OH)_4_]^−^_0.7_ was obtained from BYK Additives. Nano‐hydroxyapatite (nHA), chemical formula of Ca_10_(PO_4_)_6_·2(OH) with an average size of 20–70 nm and a specific surface area of 110 m^2^ g^−1^ were purchased from Berkeley Advance Biomaterials (Berkeley, CA) (BABI‐HAP‐N20‐A). Nano‐whitlockite (nWH), with the formula Ca_18_Mg_2_(HPO_4_)_2_(PO_4_)_12_ was generously provided by Dr. Hae Lin Jang (Harvard University). Silica nanopowder (nSiO_2_), chemical formula SiO_2_, was purchased from Sigma Aldrich.

### Nanomaterial Characterization

Validation of nanomaterials was done by evaluating chemical composition, size, morphology, and crystal structure. X‐ray diffraction (XRD) (Bruker D8 Advanced) was used to evaluate the crystalline structure of nanomaterials. A copper source for XRD was used on powdered nanomaterials that were flash‐frozen and then lyophilized. Transmission electron microscopy (TEM) was performed to evaluate the morphology and size of the nanomaterials. For this experiment, a dilute aqueous suspension (1 µg mL^−1^) of each nanomaterial was deposited onto a 400 mesh F/C carbon grid. Morphology was determined with TEM at an accelerating voltage of 200 kV using a JEOL‐JEM 2010 (Japan). Zeta potential and hydrodynamic size were measured with a Zetasizer Nano ZS (Malvern Instrument) with a He–Ne laser at room temperature (25°C). A 1 mg mL^−1^ stock solution of all nanoparticles served as the starting point. Sterile particles were obtained through utilization of a 0.2 µm filter for nSi and nSiO_2_. Sterile particles of nHA and nWH were obtained by ethanol‐wash and probe sonication to prevent particle agglomeration and loss in a 0.2 µm filter. The nanoparticle suspensions were then diluted down 1:10 000 in sterile water and used for measurement. For protein absorption experiments, 50 µg mL^−1^ solutions of various nanomaterials were incubated in media containing 10% FBS for 6 h. After incubation, the particles were washed using centrifugal filters (100 kDa MWCO) to remove loosely bound protein and resuspended in PBS. Total protein absorbed by each nanoparticle was evaluated using the MicroBCA Protein Assay kit (Catalog #23 235, Thermo Fisher).

### Cell Culture

Human mesenchymal stem cells (hMSCs) were obtained from the iliac crest of three different adult donors. These primary cells were obtained from ATCC (Catalog #PCS‐500‐012) and Rooster Bio (Catalog # MSC‐003; Lots 310 277 and 310 238) and were not modified in any way before use. The cells showed positive expression for CD29, CD44, CD73, CD90, CD105, and CD166, while the expression for CD14, CD34, CD19, and CD45 was negative. These hMSCs were cultured in alpha minimal essential medium (α‐MEM) (HyClone, GE Sciences) supplemented with 16.5% (v/v) FBS (Atlanta Biologicals) and 1% (v/v) penicillin/streptomycin (100U/100 ug/mL; Gibco). Every 2–3 days, culture media was removed and replaced with fresh media. Cells were passaged utilizing 0.5% trypsin‐EDTA once approximately 70% confluent and seeded at approximately 10000 cells per cm^2^. In vitro testing was performed on cell populations below P5. Cells were treated with and without nanomaterial (nSi, nHA, nWH, and nSiO_2_) solution (50 µg mL^−1^) and cultured for 21 days.

### Cytocompatibility Assay

Metabolic activity was evaluated via 2‐(4,5‐dimethylthiazolyl‐2)−2,5‐diphenyltetrazolium bromide (MTT) assay, according to manufacturer's protocol (ATCC). Cell cycle analysis was performed with the BD Accuri CG flow cytometer and a propidium iodide (PI) (40 µg mL^−1^) stain with RNase (100 µg mL^−1^) following earlier protocol.^[^ ^39^ ^]^ Briefly, cells were serum‐starved (0% FBS in media) for 10 h to synchronize cell populations before treatment with various nanomaterials. After 7 days of exposure, cells treated with various nanomaterials were trypsinized and fixed with −20°C ethanol. Cell pellets were washed with PBS and then incubated in PI stain for 30 min at 37°C. Cells were stored at 4°C until analysis with the BD Accuri C6 flow cytometer.

### Western Blot Analysis

For western blot, proteins were isolated after 14 days with RIPA Buffer (Catalog# J63306.AK,Thermo Fisher) from hMSCs cultured in osteoconductive (normal growth media supplemented with 10 mm β‐glycerophosphate (Sigma‐Aldrich) and 50 µm ascorbic acid (BDH Chemicals)) media with and without nanomaterial treatment (50 µg mL^−1^) and compared to an osteoinductive (osteoconductive media supplemented with 100 nm dexamethasone) control. Protein samples were separated via gel electrophoresis (Invitrogen, Mini Gel Tank) and gels were then transferred (Invitrogen, iBlot 2) to a nitrocellulose membrane per manufacture protocol. Membranes were blocked with 5% BSA in PBST (1X PBS with 0.1% Tween20) for 30 min prior to antibody staining. β‐actin (Catalog # PA5‐72633), ALPL (Catalog # MA5‐24845), type 1 collagen (COL1A1) (Catalog #PA5‐86949), and RUNX2 (Catalog #PA5‐86506) primary antibodies were purchased from ThermoFisher, secondary HRP conjugated antibodies were purchased from Boster Bio (Catalog #BA1054), and incubation was performed following manufacture protocols. Membranes were developed (SuperSignal West Pico PLUS Chemiluminescent Substrate, ThermoFisher) and imaged using LI‐COR 3600 C‐Digit Blot Scanner. LI‐COR software was used to quantify protein bands. Restoration and re‐blocking with 5% BSA in PBST of the membranes was then done for further protein analysis.

### Semi‐Quantitative ELISA Analysis

For ELISA, proteins were isolated after day 7 and day 14 using RIPA buffer as outlined above with and without nanomaterial treatment. The total protein content extracted from each sample was quantified using MicroBCA Protein Assay Kit (Catalog #252 323, Thermo Fisher). Equal amounts of protein were loaded into each well (20 µg mL^−1^) of a Nunc MaxiSorp ELISA well plate (Catalog #44‐2404‐21, Thermo Fisher) and incubated overnight at 4 degrees. Post incubation, the excess unbound antigen was removed with multiple PBS washes, blocked with SuperBlock PBS blocking buffer (Catalog #37 515, Thermo Fisher) for 30 min and washed multiple times again with PBS. Primary antibody incubation was carried out following manufacturer's instructions against GAPDH (Catalog #60004‐1‐Ig, Proteintech), RUNX2 (Catalog #RUNX2‐201AP, Thermo Fisher), OPN (Catalog #PA1‐25152, Thermo Fisher) and BMP2 (Catalog #PA1‐31215, Thermo Fisher) targets. Secondary HRP conjugated antibody (Catalog #BA1054, Boster Bio) incubation was performed following manufacture protocols. Signal development was performed using the 1‐Step Ultra TMB‐ELISA Substrate solution (Catalog #34 028, Thermo Scientific) and readings were taken at 450 nm using the Cytation 5 cell imaging multimode reader (Agilent). All experiments were performed in triplicates (n = 3) and repeated in at least two different lots of hMSCs (MSC‐003, Lot #0238 and 310 277) to ensure observations were consistent across different hMSC donors.

### hMSC Differentiation

For osteogenic differentiation studies, hMSCs were similarly seeded in 24 well‐plates at a density of 5000 cells cm^−2^. After reaching 70% confluency, cells were treated with osteoconductive media (normal growth media supplemented with 10 mm β‐glycerophosphate (Sigma‐Aldrich) and 50 µm ascorbic acid (BDH Chemicals) and the various inorganic nanomaterials (50 µg mL^−1^). For the duration of the study, media was changed every 3–4 days and replenished with fresh osteoconductive media and nanomaterial treatments at 50 µg mL^−1^. To analyze osteogenic differentiation, alkaline phosphatase (ALP) staining and kinetic activity were monitored along with matrix mineralization and quantification. First, hMSCs were fixed with 2.5% glutaraldehyde for 15–20 min. At 14 days, ALP staining was done using NBT/BCIP 1‐steps solution (Nitroblue tetrazolium/5‐Bromo‐4‐chloro‐3‐indolyl phosphate; Catalog #34 042, Thermo Fisher) for 30–60 min at room temperature. For quantification of ALP activity, hMSCs were incubated with alkaline phosphatase yellow (Sensolyte pNPP ALP assay kit, Catalog #AS‐72146, AnaSpec). Using an automated plate reader (Tecan), ALP activity as a function of pNPP metabolism (ΔOD405) was measured and activity was normalized to cell DNA content (CyQUANT, Catalog #C7026 Thermo Fisher). After 1 and 21 days, Alizarin Red staining (ARS; Electron Microscopy Sciences) was performed. The bound ARS, which was proportional to calcified matrix, and was quantified by dissolution in acetic acid (10%), neutralized by ammonium hydroxide (10%), and then measured in an automated plate reader (ΔOD405; Tecan). Both ALP and mineralized matrix were visualized with a stereomicroscope (Zeiss).

### Pathway Inhibition Studies

Osteogenic pathway inhibition of matrix mineralization was performed after 14 days using Alizarin Red staining as previously described. Canonical Wnt inhibition was performed by treating hMSCs with 10 µm of cardamonin (Catalog #18 310, Cayman Chemical) every media change (3‐4 days). BMP signaling was inhibited using 5 µm treatments of LDN‐193189 (Catalog #11 802, Cayman Chemicals) every media change. MEK1/2 was inhibited by treating with PD185342 at 5 µm (Abcam) every media change.

### Quantitative Polymerase Chain Reaction

Quantitative Polymerase Chain Reaction (qPCR) was performed on known endochondral differentiation markers (*RUNX2, IBSP, SPP1/OPN, OCN*) after treatment with nanoparticles over 7, 14, and 21‐day culture periods. Total mRNA was extracted from 6 well‐plates (seeding density: 7500 cells cm^−2^) using the Directzol RNA Microprep kit (Catalog #R2061, Zymo Research) following manufacturer's instructions. cDNA synthesis was performed using the qScript cDNA supermix (Catalog# 95048‐025, QuantaBio). qPCR was performed on 2 ng of cDNA using the primer sequences listed below (Thermo Fisher):

IBSP_F: 5′‐AACAAGGCATAAACGGCACCAGTA‐3′
IBSP_R: 5′‐CGGTAATTGTCCCCACGAGGTT‐3′
RUNX2_F:5′‐GACACCACCAGGCCAATC‐3′
RUNX2_R: 5′‐AGAACAAGGGGGCCGTTA‐3′
OCN_F: 5′‐CAAAGGTGCAGCCTTTGT GTC‐3′
OCN_R: 5′‐TCACAGTCCGGATTGAGCTCA‐3′
SPP1_F: 5′‐TGAAACGAGTCAGCTGGATG‐3′
SPP1_R: 5′‐TGAAATTCATGGCTGTGGAA‐3′
GAPDH_F: 5′‐CAGCGACACCCACTCCTC‐3′
GAPDH_R: 5′‐TGAGGTCCACCACCCTGT‐3′

### mRNA Extraction and Sample Preparation

For mRNA extraction, cells were cultured until 65% confluent and subjected to two different media compositions for 21 days. One group of cells was cultured with osteoconductive media (10 mm β‐glycerophosphate (Sigma–Aldrich) and 50 µm ascorbic acid (BDH Chemicals)) as a negative control (two technical replicates); another group was treated with nSi (50 µg mL^−1^) in osteoconductive media (two technical replicates); another group was treated with nHA (50 µg mL^−1^)in osteoconductive media (two technical replicates); another was treated with nWH (50 µg mL^−1^) in osteoconductive media (two technical replicates); another with nSiO_2_ (50 µg mL^−1^) in osteoconductive media (two technical replicates); after which media changes were performed every 3–4 days until day 21. On day 21, cells were washed with PBS and pelleted. RNA was isolated and collected via a Zymogen, High Purity RNA Isolation kit following the manufacturer's protocol. Quality of nucleic acids (1 µg) was evaluated using spectrometer absorbance ratios between 280/260 nm around 2.0.

### Library Preparation, RNA‐seq and Data Processing

The high‐output HiSeq platform was used to analyze RNA samples with TruSeqRNA sample preparation and single‐end read length of 125 bases (Charlie Johnson, Genomics and Bioinformatics Service, Texas A&M AgriLife Research, College Station, TX). The RNA‐seq aligner Spliced Transcripts Alignment to a Reference (STAR) was used to align sequenced reads to the human reference genome (hg38).^[^ ^15^ ^]^ The Reference sequence (RefSeq) genome annotation of the human genome (hg38, GRCh37 Genome Reference Consortium Human Reference 37) was used to find the gene definition, and was obtained from the University of California, Santa Cruz, genome browser. For the negative control group, 23064842 (20783935 uniquely mapped) and 33923628 (30981658 uniquely mapped) were aligned to the genome for the two replicates. For the nSi treatment group, 17700673 (16256612 uniquely mapped) and 26835751 (24998082 uniquely mapped) successfully aligned to the genome for the two replicates. For the nHA treatment group, 26241065 (24336904 uniquely mapped) and 25008428 (23023205 uniquely mapped) successfully aligned to the genome for the two replicates. For the nWH treatment group, 37357067 (33085243 uniquely mapped) and 30051222 (27655430 uniquely mapped) successfully aligned to the genome for the two replicates. For the nSiO_2_ treatment group, 26285245 (24293862 uniquely mapped) and 22002435 (20464135 uniquely mapped) successfully aligned to the genome for the two replicates. Only uniquely mapped reads were used in further analyses.

### Differential Gene Expression Analysis

Gene models were made by using the Bioconductor package Genomic Features in the R environment.^[^ ^48^ ^]^ To determine gene expression, uniquely mapped reads associated with coding exons were counted and normalized by gene length in fragments per kilobase per million (FPKM), and genes were considered expressed if present in both replicates and above 1 FPKM. Genes expressed in only one condition were considered differentially expressed. Differentially expressed genes identified by generalized linear models (GLMs) were modeled as a negative binomial distribution.^[^ ^49^ ^]^ The Bioconductor package DESeq2 was used to do this.^[^ ^16^ ^]^ R was used to perform all the analyses.

### Gene Ontology Enrichment Analysis

GO Enrichment analysis was performed using the GOStats^[^ ^50^ ^]^ Bioconductor^[^ ^51^ ^]^ package. GOStats was used to perform hypergeometric tests and generate significant terms within the biological processes (BP) ontology. Parameters for this analysis included a DEG cut‐off of FDR p‐adj < 0.05 and conditional hypergeometric test of over‐representation against the annotation package org.Hs.eg.db. We used REVIGO^[^ ^52^ ^]^ to refine the long list of significant GO terms, which decreases functional redundancies and groups terms based on semantic similarity measures. Cytoscape,^[^ ^53^ ^]^ GeneMANIA,^[^ ^54^ ^]^ and ClueGO^[^ ^55^ ^]^ were used to visualize gene networks by direct comparison to the human genome.

### Gene Set Enrichment Analysis

Gene Set Enrichment Analysis (GSEA) analysis was utilized to obtain targeted enrichment analyses of DEGs. The Molecular Signature Database (MsigDB) v7.2 was used as a reference database.^[^ ^28^ ^]^ To perform GSEA analysis, pre‐ranked lists were generated by multiplying DEGs p‐adjust values by −log_10_ and the sign of each DEG's log_2_ fold change. The ranked list ordered DEGs from most significant and positive to most significant and negative. Then, GSEA was run on the pre‐ranked lists under default settings. Gene set sizes of greater than 500 and less than 15 were excluded. The ranked lists were compared to a priori gene sets within the version c2 curated collection from the Molecular Signature Database (MsigDB v7.2) (https://data.broadinstitute.org/gsea‐msigdb/msigdb/release/6.2/c2.all.v6.2.symbols.gmt). We graphed the top 20 plots for both positive and negative GSEA gene set results. Gene sets with an FDR < 0.1 were considered significant and used in future analysis.

GSEA results were used in conjunction with Cytoscape for gene set networks.^[^ ^23^, ^47^ ^]^ To do so, GSEA software (version 4.1.0) was used to perform enrichment map visualization in conjugation with Cytoscape (version 3.8.0).^[^ ^53^ ^]^ Gene set networks were arranged in Cytoscape using the Enrichment Map plug‐in.^[^ ^56^ ^]^ Briefly, in the “GSEA” analysis setting, the pre‐ranked lists, positive and negative enrichment reports, and gene set. gmt files were loaded to build the network. The “Data Set Edges” and the “Connectivity” options were set to default settings. Gene sets included in GSEA network analysis had an FDR p‐adj < 0.1. Node cutoff was set at an FDR p‐adj of < 0.1. The “Edge Cutoff (Similarity)” was set to > 0.375. Edge weight represents the degree of similarity (number of shared genes between gene sets). Nodes were stylized to show the designated colors of treatment group which regulate gene sets, and do not represent positive or negative normalized enrichment scores (NES). Node size represents gene set size.

### Gene Set Overlap Network

GSEA results of nSi samples returned the significantly (FDR < 0.1) positively correlated gene sets Pramoonjago: Sox4 Targets Up and PID: HIF1 TFPathway. The lists of these genes were imported into GeneMANIA^[^ ^45^ ^]^ and individual networks were created. The two individual networks were merged based on gene name and genes not considered core‐enrichments were omitted. A circular layout was used to differentiate genes originating from Pramoonjago: Sox4 Targets Up, genes from PID: HIF1 TFPathway, and genes shared between the two gene sets. Nodes were stylized to show the degree of the positive sign change, where dark red represents a strong, positive significant −log_10_ p‐adj through GSEA analysis. Genes stylized in black italics met a DEG cut off of p‐adj < 0.05. Edges in grey represent genes found in a priori gene sets to be co‐expressed together. Edges in red with arrow heads represent pathway interactions based on known pathway interactions from the PID: HIF1 TFPathway.

### Statistical Analysis

Statistical analysis was performed in GraphPad Prism. One‐way analysis of variance (ANOVA) coupled with Tukey's post‐hoc were performed. Plots were graphed as mean and standard deviation and statistical significance was presented as ^*^p‐value < 0.05, ^**^p‐value < 0.01, ^***^p‐value < 0.001, and ^****^p‐value < 0.0001.

## Conflict of Interest

The authors declare no conflict of interest.

## Author Contributions

A.K.G. and I.S. conceived the original idea of the project and provided funds, instruments, and supervised the project. A.M., A.M.B., and L.M.C. lead study design, performed experiments, analyzed data, and prepared the manuscript. A.M. led the revision of the manuscript and contributed to validation of multiple hypothesis identified from RNA‐seq and addressed the reviewers comments. L.M.C. performed preliminary in vitro studies and RNA‐seq sample preparation. A.M.B. analyzed the RNA‐seq data and performed initial framework of this work by leading hMSCs differentiation studies. M.K.J. performed materials characterization including TEM and XRD. A.L.K. and I.S. performed computational analysis. All the authors commented on the results and participated in the writing and critical revision of the manuscript.

## Supporting information

Supporting Information

## Data Availability

The data that support the findings of this study are openly available in zenodo at https://doi.org/10.5281/zenodo.10719296, reference number 10719296.
