# Inorganic Biomaterials Shape the Transcriptome Profile to Induce Endochondral Differentiation

**DOI:** 10.1002/advs.202505203

**Published:** 2025-04-26

Aparna Murali, Anna M. Brokesh, Lauren M. Cross, Anna L. Kersey, Manish K. Jaiswal, Irtisha Singh^*^ and Akhilesh Gaharwar^*^

Volume11, Issue29 August 7, 2024 2402468

DOI 10.1002/advs.202402468

In the original published article, the data availability statement was incomplete. Here is the complete Data Availability Statement. The conclusion remains unaffected by the correction. The authors apologize for any inconvenience this may have caused.

**Data Availability Statement**

The data that support the findings of this study are openly available in Zenodo at https://doi.org/10.5281/zenodo.10719296. RNA‐seq data reported here have been deposited in the Gene Expression Omnibus (GEO) database (accession no. GSE193172 (hMSCs, nSi) and GSE193173 (nHA, nWH, and nSiO2) (www.ncbi.nlm.nih.gov/geo). The experimental method presented in this manuscript was adapted from our previously published work (Brokesh et al., Science Advances 8(17) eabl9404 (2022), DOI: 10.1126/sciadv.abl9404).

