# Supplementary material for: Inorganic Biomaterials Shape the Transcriptome Profile to Induce Endochondral Differentiation
Source: Adv Sci (Weinh). 2024 May 13;11(29):2402468. doi: 10.1002/advs.202402468 (PMC11304299; doi:10.1002/advs.202402468)
Supplement: Supplementary file 1 — Supporting Information [file ADVS-11-2402468-s001.pdf]

ADVANCED SCIENCE
Open Access

Supporting Information

for *Adv. Sci.*, DOI 10.1002/advs.202402468

Inorganic Biomaterials Shape the Transcriptome Profile to Induce Endochondral Differentiation

Aparna Murali, Anna M. Brokesh, Lauren M. Cross, Anna L. Kersey, Manish K. Jaiswal, Irtisha Singh and Akhilesh Gaharwar**

Supporting Information

Inorganic biomaterials shape the transcriptome profile to induce endochondral differentiation

Aparna Murali[1], Anna M. Brokesh[1], Lauren M. Cross[1], Anna L. Kersey[1], Manish K. Jaiswal[1], Irtisha Singh[1,4], Akhilesh Gaharwar[1,2,3,5*]*

Aparna Murali, A. M. Brokesh, L. M. Cross, A. L. Kersey, Dr. M. K. Jaiswal, Prof. I. Singh, Prof. A. K. Gaharwar
Department of Biomedical Engineering, College of Engineering, Texas A&M University, College Station, TX 77843, USA

Prof. I. Singh
Department of Cell Biology and Genetics, College of Medicine, Texas A&M University, Bryan, TX 77807-3260, USA

Prof. I. Singh, Prof. A. K. Gaharwar
Interdisciplinary Program in Genetics and Genomics, Texas A&M University, College Station, TX 77843, USA

Prof. A. K. Gaharwar
Department of Material Science and Engineering, College of Engineering, Texas A&M University, College Station, TX 77843, USA

*Corresponding author E-mail: gaharwar@tamu.edu (AKG); isingh@tamu.edu (IS)

Keywords: Regenerative medicine, stem cells, RNA-seq, biomaterials, nanomaterials.

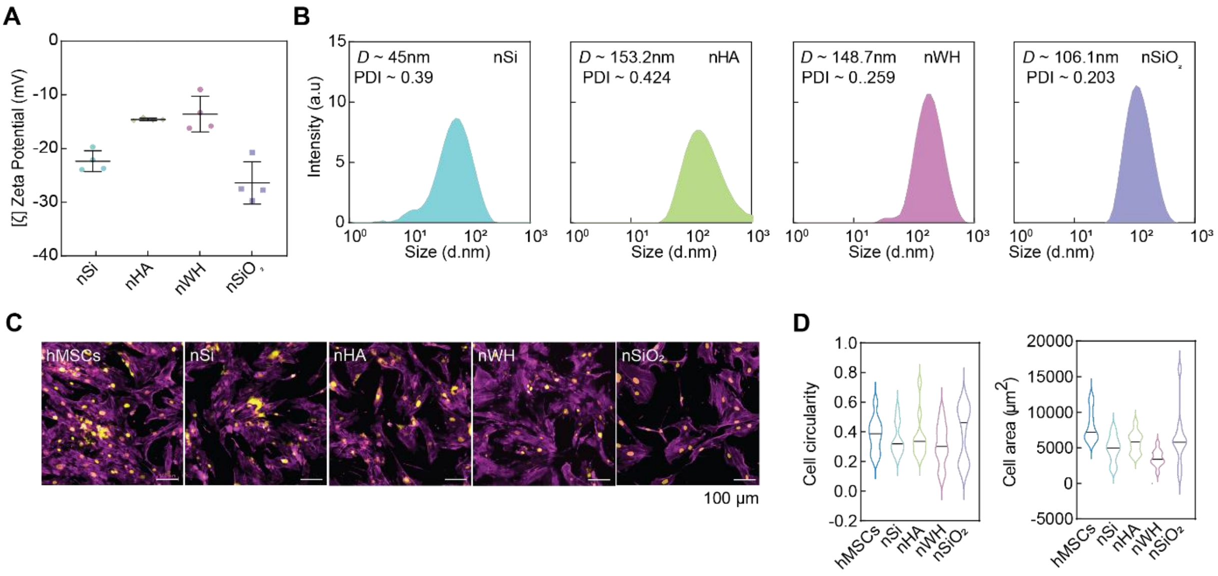

Figure S1. Physiochemical characteristics of inorganic nanomaterials.

(A) Zeta potential (ζ) shows the charged nature and stable aqueous nature of suspended nanomaterials. The zeta-potential (ζ) shows nSi has a charge of -22.4 mV, nHA; -14.6 mV, nWH; -13.6 mV, and $nSiO_2$; 26.4 mV.

(B) Dynamic Light Scattering (DLS) shows the hydrodynamic diameter (D_h) in aqueous conditions and polydispersity index (PDI) of nanomaterials is as follows; nSi; D_h: ~45nm, PDI: ~0.39, nHA; D_h: ~153.2nm, PDI: ~0.424, nWH; D_h: ~148.7nm, PDI: ~0.259, nSiO2; D_h: ~106.1nm, PDI: ~0.203.

(C) Qualitative effect of nanomaterials on hMSCs morphology. Cytosketal is shown in pink and nucleus is shown in yellow.

(D) Cell circularity and cell area are calculated from corresponding cytoskeletal images for various inorganic nanomaterial treatments

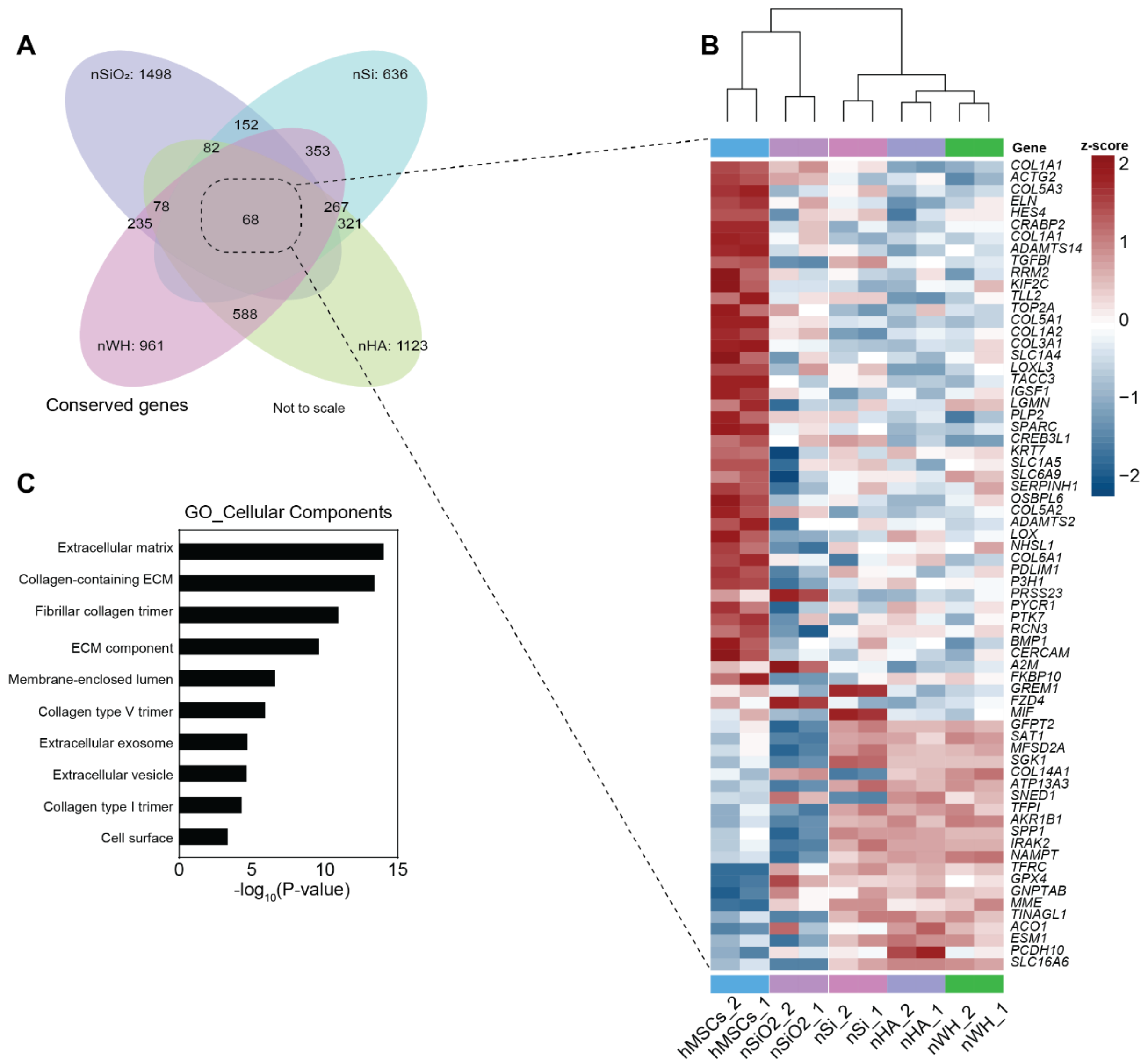

Figure S2. Nanomaterials perturb similar differentially expressed genes (DEGs) (*p-adj* < 0.05) between treatments.

A) 68 DEGs are shared between all of the treatment groups, but nHA and nWH had the most shared perturbed genes (588) compared to other nanomaterials.

B) Hierarchical clustering of 68 common genes in hMSC samples treated with (nSi, nHA, nWH and $nSiO_2$) and without nanomaterials (hMSCs) based on mRNA expression obtained from RNA-seq. The heatmap shows the differentially expressed genes (Log_2FPKM of DEGs, *p-adjust* < 0.05) across all treatment groups

C) Gene ontology analysis of 68 common genes against a background list of all DEGs (*p-value* < 0.05). Cellular component subset represented formation of collagen extracellular matrix and endocytic transport of inorganic nanomaterials.

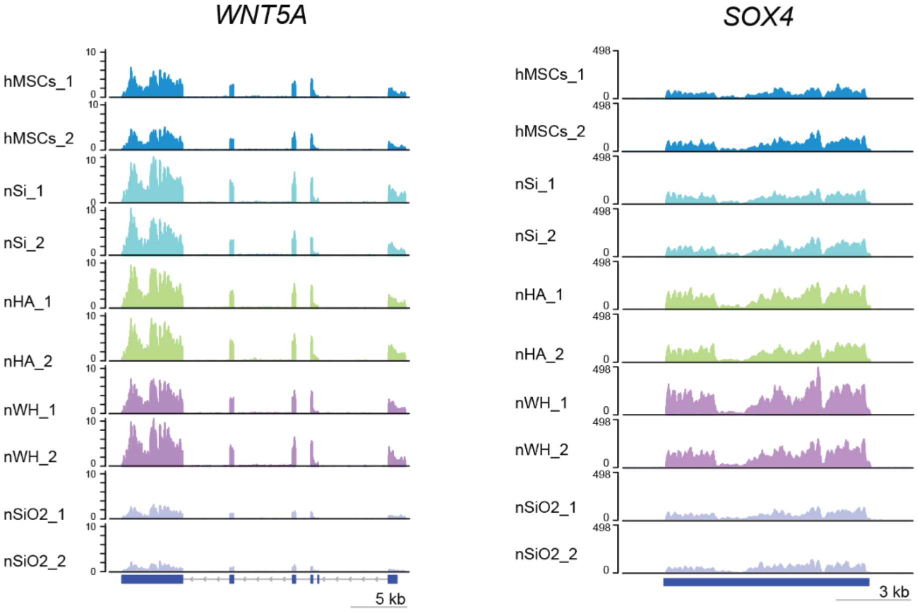

Figure S3. RNA-seq tracks showing normalized mRNA expression (aligned reads normalized by total library size- transcript per million (TPM) at the genomic locus of Wnt family member 5A (*WNT5A*), and SRY-Box transcription factor 4 (*SOX4*) for each treatment group (nSi, nHA, nWH, $nSiO_2$).

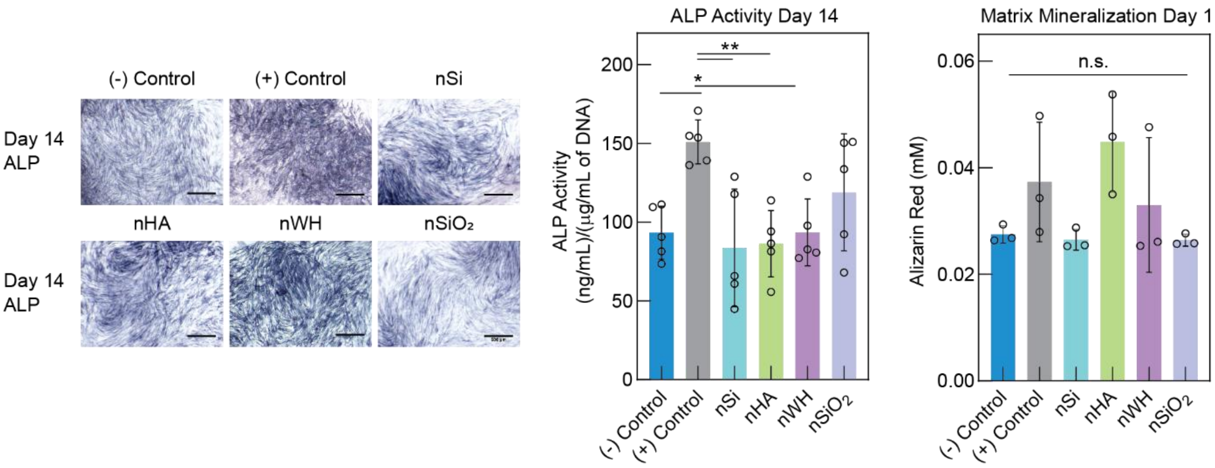

Figure S4. ALP activity and matrix mineralization was evaluated for nanomaterials at day 14 and day 1, respectively. ALP production was qualitatively assessed via staining, and the activity was assessed via PnPP enzymatic assay. Matrix mineralization was assessed using Alizarin Red Staining (ARS). Statistical comparison between samples was performed using one-way ANOVA and Dunnett's multiple comparison test, with a single pooled variance. (ns= not significant, *$p < 0.05$, **$p < 0.01$)

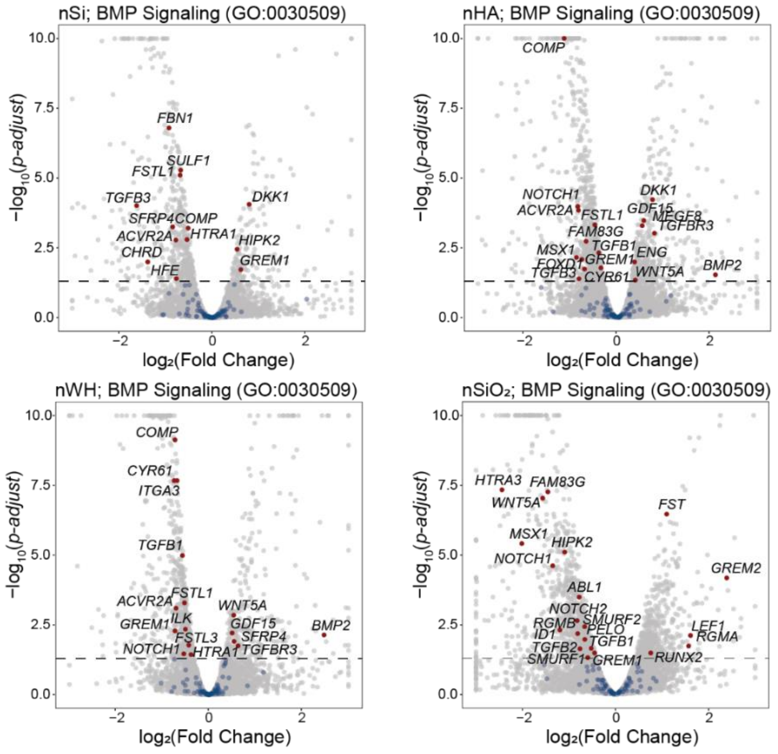

Figure S5. Volcano plots showing DEGs associated with BMP signaling (GO:0030509). The graphs show -$\log_{10}$(*p-adj*) significance values of nSi, nHA, nWH, and $nSiO_2$ DEGs (*p-adj* < 0.05) associated with BMP signaling (GO:0030509), a pathway known to be associated with endochondral ossification, plotted based on $\log_2$(fold change).
